# Cleanroom‐Free Direct Laser Micropatterning of Polymers for Organic Electrochemical Transistors in Logic Circuits and Glucose Biosensors

**DOI:** 10.1002/advs.202307042

**Published:** 2024-01-15

**Authors:** Alessandro Enrico, Sebastian Buchmann, Fabio De Ferrari, Yunfan Lin, Yazhou Wang, Wan Yue, Gustaf Mårtensson, Göran Stemme, Mahiar Max Hamedi, Frank Niklaus, Anna Herland, Erica Zeglio

**Affiliations:** ^1^ Department of Micro and Nanosystems KTH Royal Institute of Technology Malvinas väg 10 Stockholm 100 44 Sweden; ^2^ Synthetic Physiology lab Department of Civil Engineering and Architecture University of Pavia Via Ferrata 3 Pavia 27100 Italy; ^3^ Division of Nanobiotechnology SciLifeLab Department of Protein Science KTH Royal Institute of Technology Tomtebodavägen 23a Solna 171 65 Sweden; ^4^ AIMES – Center for the Advancement of Integrated Medical and Engineering Sciences Department of Neuroscience Karolinska Institute Stockholm 17177 Sweden; ^5^ Guangzhou Key Laboratory of Flexible Electronic Materials and Wearable Devices School of Materials Science and Engineering Sun Yat‐sen University Guangzhou 510275 P. R. China; ^6^ Key Laboratory for Polymeric Composite and Functional Materials of Ministry of Education School of Materials Science and Engineering Sun Yat‐sen University Guangzhou 510275 P. R. China; ^7^ Mycronic AB Nytorpsvägen 9 Täby 183 53 Sweden; ^8^ Department of Fibre and Polymer Technology School of Engineering Sciences in Chemistry Biotechnology and Health KTH Royal Institute of Technology Teknikringen 56 Stockholm 10044 Sweden; ^9^ Wallenberg Initiative Materials Science for Sustainability Department of Materials and Environmental Chemistry Stockholm University Stockholm 114 18 Sweden

**Keywords:** conjugated polymer, direct writing, organic electrochemical transistor, poly(3,4‐ethylenedioxythiophene) polystyrene sulfonate, ultrashort pulsed lasers

## Abstract

Organic electrochemical transistors (OECTs) are promising devices for bioelectronics, such as biosensors. However, current cleanroom‐based microfabrication of OECTs hinders fast prototyping and widespread adoption of this technology for low‐volume, low‐cost applications. To address this limitation, a versatile and scalable approach for ultrafast laser microfabrication of OECTs is herein reported, where a femtosecond laser to pattern insulating polymers (such as parylene C or polyimide) is first used, exposing the underlying metal electrodes serving as transistor terminals (source, drain, or gate). After the first patterning step, conducting polymers, such as poly(3,4‐ethylenedioxythiophene):poly(styrene sulfonate) (PEDOT:PSS), or semiconducting polymers, are spin‐coated on the device surface. Another femtosecond laser patterning step subsequently defines the active polymer area contributing to the OECT performance by disconnecting the channel and gate from the surrounding spin‐coated film. The effective OECT width can be defined with high resolution (down to 2 µm) in less than a second of exposure. Micropatterning the OECT channel area significantly improved the transistor switching performance in the case of PEDOT:PSS‐based transistors, speeding up the devices by two orders of magnitude. The utility of this OECT manufacturing approach is demonstrated by fabricating complementary logic (inverters) and glucose biosensors, thereby showing its potential to accelerate OECT research.

## Graphical abstract

Ultrafast focused femtosecond laser has been introduced for the direct micropatterning of organic electrochemical transistors (OECTs), providing high resolution (2 µm), selective cleanroom‐free patterning of insulating and conjugated polymer layers while preserving device operation, and high flexibility in device design. The approach has been validated in the fabrication of complementary inverters and glucose biosensors.

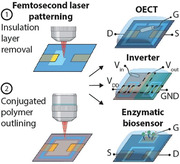

## 1 Introduction

Bioelectronics aims to develop devices for a wide range of applications, including monitoring tissues/cells in vivo and in vitro, diagnosing diseases in point‐of‐care testing, and detecting chemical or biological species.^[^ ^1^, ^2^, ^3^, ^4^, ^5^, ^6^, ^7^, ^8^, ^9^ ^]^ These applications require devices with high selectivity and sensitivity, such as the organic electrochemical transistor (OECT). OECTs are three‐terminal electronic devices where the channel material is an organic mixed ionic/electronic conductor (OMIEC) connecting the source and drain electrodes.^[^ ^10^ ^]^ The gate electrode is electrically coupled to the channel through an electrolyte solution. The nature of the electrolyte depends on the application, but aqueous electrolyte solutions are the choice for applications requiring contact with biological fluids, such as cell culture media for in vitro electrophysiology and fluids containing the analyte of interest for biosensors. The OECT working mechanism relies on redox‐based modulation of the channel conductivity. Upon application of a gate bias, electrolyte ions penetrate the channel volume, changing the redox state and conductivity of the OMIEC (typically a conjugated polymer), resulting in high signal amplification that can be exploited in sensing.^[^ ^11^, ^12^ ^]^ Both p‐type (holes as charge carriers) and n‐type (electrons as charge carriers) conjugated polymers have been used to fabricate OECTs. Recently, the integration of p‐ and n‐type OECTs has enabled the fabrication of complementary circuits, a key step to advancing the sophistication of OECT‐based technologies and providing unprecedented signal amplification. ^[^ ^13^, ^14^, ^15^ ^]^

In contrast to inorganic semiconductors used in conventional electronics, conjugated polymers offer ease of processing from solution under near‐ambient conditions, providing the possibility to implement time‐ and cost‐effective fabrication methods, including screen printing,^[^ ^16^, ^17^ ^]^ aerosol printing,^[^ ^18^ ^]^ inkjet printing,^[^ ^19^ ^]^ capillary printing,^[^ ^20^ ^]^ spray coating,^[^ ^15^ ^]^ filter‐based gel micromachining,^[^ ^2^ ^]^ and 3D printing.^[^ ^21^, ^22^ ^]^ While providing fast and scalable layer deposition and patterning, these approaches reach minimum pattern features in the range of tens of micrometers.^[^ ^21^, ^23^ ^]^ Thus, these techniques are not suited for applications requiring microfabricated OECT channels with micrometer or sub‐micrometer resolution, such as for single‐cell recordings, electro‐anatomical mapping, miniaturized microfluidic sensors, and ultrafast neuromorphic switching.^[^ ^5^, ^10^, ^24^, ^25^, ^26^ ^]^ Additionally, all these printing techniques require process optimization for every ink formulation, thereby providing a barrier to versatile and efficient fabrication, fast prototyping, and material innovation.^[^ ^21^ ^]^ For high‐resolution applications, cleanroom‐based lift‐off processes are currently the standard approach since they reliably provide channel dimensions as small as 0.5 µm independently of the used channel material.^[^ ^27^, ^28^, ^29^, ^30^, ^31^, ^32^ ^]^ Cleanroom‐based polymer patterning, however, relies on complex and time‐demanding multiple‐step processes that must be carried out within an expensive cleanroom environment and involve solvent and developer baths that increase the environmental impact of device fabrication.^[^ ^33^, ^34^, ^35^ ^]^

In addition to lift‐off, wet chemical processing was recently introduced to decrease the number of fabrication steps required for OECT fabrication.^[^ ^32^ ^]^ This process relies on the deposition of a photoresist followed by channel opening via photolithography, spin‐coating of the conducting polymer, and exposure to a solvent for resist removal (e.g., dimethyl sulfoxide). For poly(3,4‐ethylenedioxythiophene) polystyrene sulfonate (PEDOT:PSS), the loss of film thickness upon solvent exposure over time was prevented by adding (3‐glycidyloxypropyl)trimethoxysilane (GOPS) crosslinker. While the method could be, in principle, also applied to uncrosslinked organic semiconductors, that would require careful choice of the photoresist, which should dissolve in an orthogonal solvent with respect to the active material and be benign to the environment upon disposal (e.g., free from bioaccumulative per‐ and polyfluoroalkyl substances).^[^ ^36^ ^]^

Laser ablation was introduced in microfabrication to enable flexible pattern adjustments without requiring expensive shadow‐ or photo‐masks. However, both continuous‐wave (CW) and pulsed lasers suffer from a large heat‐affected zone in the laser‐illuminated areas, causing the decomposition of the conducting polymer.^[^ ^37^, ^38^ ^]^ Alternatively, laser ablation has been used to remove a surface passivated insulating layer, allowing successive deposition of conjugated polymer only on the depassivated area corresponding to the OECT, making this approach more useful for material testing and only posing limitation of the substrate material.^[^ ^37^ ^]^ This solution is still limited in throughput by using a continuous laser source. Ultrafast laser tools (with pulse duration below the picosecond range and peak power in the MW range) are promising for efficient and fast patterning with microscale precision.^[^ ^39^, ^40^ ^]^ Moreover, the ultrashort laser pulse duration ensures that material removal occurs through non‐thermal mechanisms such as Coulomb explosion and bond dissociation.^[^ ^41^, ^42^ ^]^ The use of femtosecond lasers was recently reported for structuring of PEDOT:PSS thin films with high‐resolution (i.e., grooves with widths as small as 4–5 µm).^[^ ^43^, ^44^, ^45^ ^]^ However, the reported repetition rates (40 kHz or lower) do not enable fast prototyping and scalable manufacturing. Moreover, the resulting pulse energy values (500 nJ or higher) do not allow for selective removal of the exposed conducting polymer layer without damaging the underlying insulating polymer layers.^[^ ^43^ ^]^

**Figure 1:**
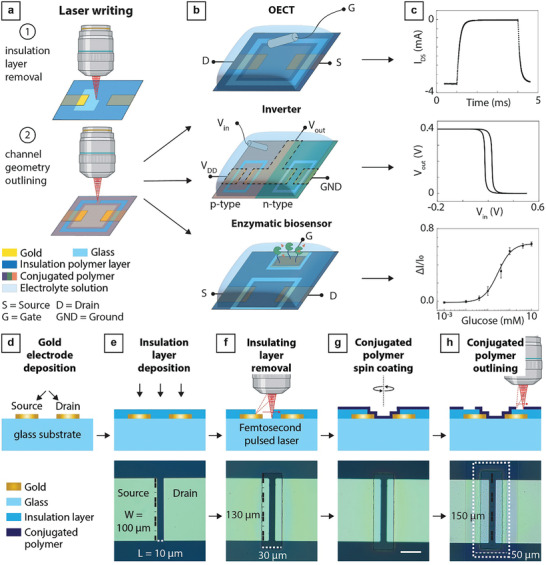
Simple and versatile OECT femtosecond laser microfabrication and examples of applications. a) Schematic overview of the OECT manufacturing process using femtosecond pulsed laser writing. The exposed polymer insulating layer is first removed by laser direct writing, exposing the underlying gold electrode contacts. After spin‐coating the polymer layer, the femtosecond pulsed laser writing is used again to pattern the thin polymer film and isolate the transistor channel from the remaining polymer film. b) Schematic overview and c) representative measurements of (from the top) realized OECTs, inverter circuits, and enzymatic biosensors, which have been manufactured using the proposed approach. d–h) Workflow of femtosecond laser microfabrication in OECT patterning: d) fabrication of the gold electrode contacts and e) deposition of the insulation polymer layer. f) Removal of the insulating polymer with femtosecond pulsed laser writing, leading to an opening of dimensions 130 µm x 30 µm and exposing the gold electrode contacts. g) Spin‐coating of the OMIEC (i.e., the OECT active material) to form a thin film on top of the sample. h) Use of the femtosecond pulsed laser to outline the area of active material contributing to the OECT performance (a 150 µm x 50 µm area marked with a white dotted line in the brightfield image). This step isolates the OMIEC area connected to the electrode contacts from the remaining polymer film. The photos show typical OECT devices. Scale bar, 25 µm.

In this work, we report direct laser writing using femtosecond laser exposure for efficient and fast microfabrication of OECT devices, OECT‐based circuits, and OECT‐based biosensors. The process employs focused femtosecond laser pulses for multiphoton ablation at the focal point of the laser beam.^[^ ^46^ ^]^ To manufacture the OECT devices, we used direct laser writing to selectively pattern the insulating polymer layer on top of gold electrodes and the conjugated polymer layer (**Figure** **1**a). This approach for subtractive micromanufacturing allows direct polymer patterning with resolution limits comparable to those of photolithography‐based patterning techniques, approaching 2 µm, but without the need for a cleanroom. Using state‐of‐the‐art laser beam scanning systems, our approach allows high throughput and scalable OECT manufacturing. The method is ideal for prototyping OECTs due to its flexibility in changing the patterning layout and its compatibility with different types of conjugated polymers. We verified the functionality of our manufacturing approach by comparing OECTs with and without a patterned/outlined conjugated polymer layer by recording significant differences in the leakage current and ON/OFF switching speed for PEDOT:PSS transistors. Finally, we demonstrated that our versatile method can be used to manufacture complementary logic (i.e., inverters) and biosensors, such as enzyme‐based glucose sensors (Figure 1b,c).^[^ ^43^, ^47^ ^]^

## 2 Results and Discussion

### 2.1 Direct Laser Writing of Conjugated Polymers to Fabricate Organic Electrochemical Transistors (OECTs)

Our proposed method enables simple and flexible manufacturing of OECTs using direct femtosecond laser writing of the insulating and active layers. This approach for polymer patterning is not dependent on photolithography‐based processes under cleanroom conditions that are otherwise needed to fabricate OECTs with single‐digit micrometer resolution. We used a commercially available high‐speed femtosecond laser lithography system (Nanoscribe GT2, 780 nm, 80 MHz, up to 100 mm s^−1^ scan speed). We started with gold electrodes on a glass substrate and deposited an insulating layer (Parylene C or polyimide) on top (Figure 1d,e; see Experimental Section for details on the substrate preparation and insulators deposition). We designed the gold electrode configuration to enable OECTs with different channel geometries and in‐plane gate electrodes, as well as the implementation of complementary inverter circuits and biosensors (Figure S1, Supporting Information).

To optimize the insulating layer removal step, we compared live microscopy feedback and microscopy images of parylene C and polyimide layers after illumination with different powers and scan speeds. In the parametric sweep, we found no laser exposure setting producing damage to the glass substrate. We observed that the removal of parylene C or polyimide was occurring efficiently only at relatively low speed (2 mm s^−1^ or lower) and high power (>50 mW). These results are in accordance with the properties of glass, parylene C, and polyimide – materials known for their stability under a wide range of temperatures and optical densities.^[^ ^48^, ^49^, ^50^ ^]^ Using suitable combinations of laser power and speed in the mentioned regime, laser exposure produces a plasma and enables the removal of the insulating polymer film, which can be appreciated using a live brightfield video feed during patterning (parylene C removal in Video S1, Supporting Information). We then imaged the exposed area to verify the removal of the insulating layer and determine the optimal parameters for the insulating polymer removal. Since burned or decomposed parylene C and polyimide can become conductive, we performed a qualitative resistance measurement with a hand multimeter to check that the patterned area were insulative between the electrodes (resistance values above 2 MΩ).^[^ ^51^ ^]^ Our optimization process showed that a line array of high‐power laser pulses of 600 pJ, a scan speed of 1 mm s^−1^, and a line distance of 250 nm are optimal to locally remove the insulating layer and open up a window without damaging the gold electrode contacts underneath (Figure 1f; Video S1, Supporting Information). Using these settings, we laser‐patterned an area of 30 µm × 120 µm in the insulating polymer layer, which is enough to expose the electrode contacts defining the OECT channel area. After patterning the insulating polymer layer, the samples were air plasma treated to remove the remaining debris from the laser writing (Figure S2, Supporting Information).

The OMIEC material was then spin‐coated from solution on top of the patterned samples (Figure 1g). To define the area of OMIEC material contributing to the OECT performance, we reused the same laser system to outline an area around the drain and source terminals. We found that pulse energies of 50% to 75% below the ablation threshold for the insulating polymer layer are sufficient to generate an outlining line around the channel area exhibiting a different color with respect to the rest of the OMIEC film (Figure 1h and Experimental section for details; Video S2, Supporting Information), possibly indicating a change in the doping state of the OMIEC. Resistance values above 2 MΩ confirmed that the outlining step destroys the OMIEC film's ability to transport charge carriers in the exposed area, electrically decoupling the portion of OMIEC above the source and drain electrode contacts from the remaining OMIEC film. Moreover, we found that, in contrast to patterning of the insulating polymer, the outlining process occurs without significant plasma formation, enabling the outlining of the OMIEC film while preserving the insulating polymer layer underneath. We patterned the active layer of OECTs by outlining a rectangle 150 µm wide and 50 µm long around the patterned area, totaling an outlined area of 7.5·10^−5^ cm^2^. Here we refer to the OECTs in which the conjugated polymer layer has been patterned as “outlined OECTs”. “Non‐outlined OECTs” will refer to samples where the OMIEC film was patterned by manually cleaning the portion outside the transistor layout area (Figure S1, Supporting Information) before annealing, leading to a rectangular non‐outlined area of ≈0.8 cm^2^.

**Figure 2:**
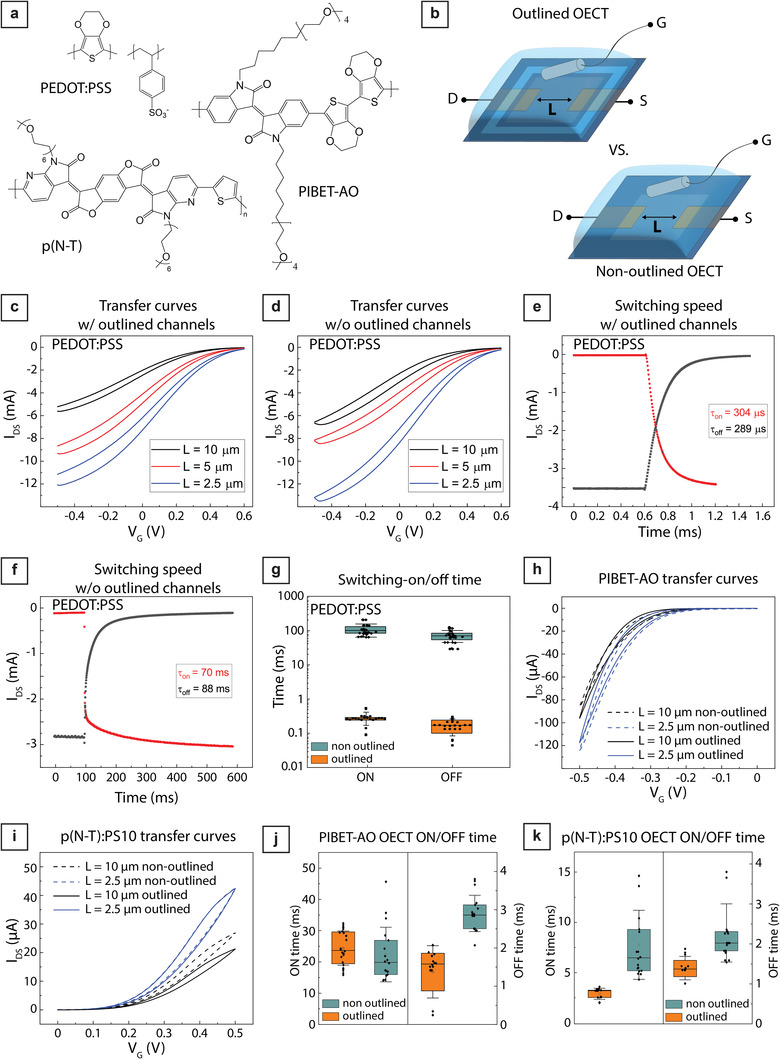
OECT characteristics of different conjugated polymers comparing outlined and non‐outlined OECTs. a) Structural formula of the three different polymers PEDOT:PSS, PIBET‐AO, and p(N‐T) used to fabricate OECTs. b) Schematic overview of the OECT measuring setup comparing outlined and non‐outlined OECTs using an Ag/AgCl pellet as the gate electrode and 0.1 M NaCl(aq) as the electrolyte. c,d) Average transfer curves for c) outlined PEDOT:PSS OECTs and d) non‐outlined PEDOT:PSS OECTs with channel lengths of 10, 5, and 2.5 µm. For averaged transfer curves, each line represents the average of five devices, V_D_ = −0.6 V. e,f) Representative switching behavior of an outlined and non‐outlined PEDOT:PSS OECT, respectively. g) Switching OFF and ON times of outlined and non‐outlined PEDOT:PSS OECTs with a channel length of 10 µm. We recorded four ON (V_G_ = 0.5 V) and four OFF (V_G_ = −0.6 V) switching events per device (eight devices in total). h,i) Average transfer curves for outlined (solid) and non‐outlined (dashed) i) p‐type accumulation mode PIBET‐AO OECTs (V_D_ = −0.5 V) and j) n‐type accumulation mode p(N‐T):PS10 OECTs (V_D_ = 0.5 V) with channel length of 10 and 2.5 µm. For averaged transfer curves, each line represents the average of five devices. j)‐k) Switching OFF and ON times of outlined and non‐outlined j) PIBET‐AO (V_G,ON_ = −0.5 V and V_G,OFF_ = 0 V) and k) p(N‐T):PS10 (V_G,ON_ = 0.5 V and V_G,OFF_ = 0 V) OECTs with a channel length of 10 µm. We recorded four ON and four OFF switching events per device (six devices in total). For g, j, and k), each dot is the duration of single switching event. Error bars show the standard deviations, with the centerline being the median value, and box plots corresponding to 25 and 75 percentiles. The transfer curves (panels c, d, h, and i) have also been plotted in log scale in Figure S4 in the Supporting Information.

We first evaluated the effectiveness of our patterning method for a standard OECT configuration consisting of an Ag/AgCl pellet as the gate electrode and 0.1 M NaCl_(aq)_ as the electrolyte.^[^ ^52^ ^]^ First, we used a commercially available conducting polymer blend (i.e., PEDOT:PSS) to realize depletion‐mode OECTs (**Figure** **2**a).

To evaluate the influence of insulator (parylene C) patterning on OECT operation and performance upon scaling, we exposed channels with a width of 100 µm and three different gap distances (channel length of the OECT) of 10, 5, or 2.5 µm between the source and drain electrode contacts. Moreover, we compared the outlined OECTs to the non‐outlined OECTs to ensure the successful outlining of the conducting polymer film (Figure 2b). Output and transfer characteristics show that all PEDOT:PSS OECTs operate in mixed mode at gate voltages (V_G_) from −0.5 V (completely ON) to 0.6 V (completely OFF), consistent with previous results on PEDOT:PSS OECT operation (Figure 2c,d; Table S1 and Figure S3a,b, Supporting Information).^[^ ^33^ ^]^ Transfer characteristics show that, in agreement with previous reports, decreasing the channel length while keeping the same thickness and width leads to an increase in maximum drain‐source current and transconductance (Figure 2c,d; Figure S3c–d, Supporting Information; see Figure S4a,b for the transfer curves in log scale in the Supporting Information).^[^ ^53^ ^]^ These data indicate that, for a constant laser‐patterned area of 30 µm × 120 µm, steady–state device performance is controlled by the effective channel area between the drain and source electrode contacts.

Outlining of the conducting polymer film did not determine any significant differences in maximum drain‐source current (I_DS_) and the magnitude and position of the maximum transconductance (g_m_) peak, located at V_G_ ≈0.1 V (Figure 2c,d; Figure S3c,d, Supporting Information). A significant change upon outlining is observed in the maximum gate current, dropping from ≈130 µA for non‐outlined to 1.6 µA for outlined devices (Figure S5a,b and Table S2, Supporting Information). Outlining PEDOT:PSS also results in large differences in the ON/OFF switching speeds of the OECTs. Non‐outlined PEDOT:PSS OECTs exhibit ON/OFF switching times around hundreds of milliseconds, whereas outlined OECTs switch ON/OFF at around hundreds of microseconds, making them three orders of magnitudes faster (Figure 2e–g; Figure S6a,b, Supporting Information). Such differences are similar across all channel sizes, indicating that the outlining step plays a major role in determining the switching speed.

To evaluate the flexibility of our approach independently of materials type, we manufactured accumulation mode p‐ and n‐type OECTs. In this case, we used polyimide as the insulation layer, to demonstrate that devices can be fabricated using insulating polymers that don´t require specialized equipment for chemical vapor deposition, as for the case of parylene C. We used the semiconducting p‐type polymer PIBET‐AO and the semiconducting n‐type polymer blend p(N‐T): 10 kDa polystyrene with a 1:6 ratio (herein referred to as p(N‐T):PS10), which were selected based on current output and device stability (Figure 2a, see Experimental section for details of the blend preparation).^[^ ^13^, ^14^ ^]^ Similar to what was observed for PEDOT:PSS OECTs, outlining accumulation mode OECTs showed no impact on the overall transfer characteristics (Figure 2h,i; Figure S5c–f and Table S3, Supporting Information). PIBET‐AO devices operate at V_D_ = −0.5 V and V_G_ from 0 to −0.5 V, while p(N‐T):PS10 devices operate at V_D_ = 0.5 V and V_G_ from 0 to 0.5 V, consistent with previous reports.^[^ ^13^, ^14^ ^]^ All devices exhibit OFF drain currents below the background noise (current level) of the measurement configuration (≈10 nA), indicating that both PIBET‐AO and p(N‐T):PS10 OECTs present high channel resistance in the OFF state (V_G_ = 0 V). Moreover, outlined and non‐outlined OECTs show similar changes in maximum device current and transconductance upon decreasing channel length (from 10 to 2.5 µm, Table S3, Supporting Information); see Figure S4c,d for the transfer curves in log scale in the Supporting Information).

Data on gate current reveal that, for accumulation mode OECTs, outlining determines a decrease in the maximum gate current of around one order of magnitude (Figure S5c–f and Table S2 in the Supporting Information). ON/OFF switching speeds showed little differences upon outlining (Figure 2j,k; Figure S6c–h, Supporting Information). The magnitude of the changes in maximum gate currents and switching times differ significantly from the comparatively larger ones observed for PEDOT:PSS‐based OECTs. The semiconducting character of PIBET‐AO and p(N‐T):PS10 is a likely contributor to this difference. In contrast to PEDOT:PSS, the semiconducting polymers provide higher resistance toward lateral ionic mobility within the polymer film. This is further supported by the electrochromic changes of the whole area in contact with the electrolyte observed for non‐patterned PEDOT:PSS films and from the maximum OFF current, which is at least 100 times larger for the depletion mode PEDOT:PSS OECTs with respect to accumulation mode OECTs.^[^ ^54^ ^]^ Nevertheless, the significant drop in maximum gate current in the outlined PIBET‐AO and p(N‐T):PS10 OECTs compared to non‐outlined OECTs indicates that the pattering method works and also improves the performance of OECTs made using semiconducting polymers (Figure 2j,k; Figure S6e–h, Supporting Information).

To further investigate the doping and de‐doping dynamics, we used a high‐speed camera and recorded the electrochromic changes in the polymer films upon doping/de‐doping through different regions of both the conducting PEDOT:PSS (Figure S7a,b and Videos S3 and S4, Supporting Information) and semiconducting PIBET‐AO (Figure S7c,d and Videos S5 and S6, Supporting Information) films during OECT switching. In line with transient responses, videos of PEDOT:PSS‐based OECTs show that gate voltage ON/OFF switches from V_G_ = 0.6 to −0.5 V lead to a considerably slower modulation of PEDOT:PSS transmission in unpatterned channels with respect to the patterned ones. In absence of an outline, we could not observe any major differences in the switching speed between the channel region and the surrounding area. Outlining confined the transmission changes of PEDOT:PSS within the channel area, with no changes observed in the surrounding PEDOT:PSS film, as expected (Figure S7b, Supporting Information). Videos of PIBET‐AO‐based OECTs show that changes in transmission within the channel area occur at a similar time regime for outlined and non‐outlined OECTs, in agreement with transient response data (upon same gate voltage switches from V_G_ = 0 V to −0.5 V). As for PEDOT:PSS, PIBET‐AO based devices show no transmission changes in areas outside the outlined OECT channels.

In both PEDOT:PSS and PIBET‐AO OECTs, we observed a spatial difference in the electrochromism, which becomes slower when moving from the channel area to the side of the devices (Figure S7b,d, Supporting Information). However, in contrast to PEDOT:PSS, PIBET‐AO film transmission changed considerably faster close to the drain than to the source, in line with the literature.^[^ ^59^ ^]^ These spatial differences in electrochromism can be explained by considering the high conductivity and volumetric capacitance of PEDOT:PSS, which determines the movement of both electronic and ionic charge carriers throughout the entire film connected to the drain and source electrodes.^[^ ^54^ ^]^ The large variation in the area of the polymer films connected to the electrodes for outlined and non‐outlined OECTs (from 7.5·10^−5^ cm^2^ and 0.8 cm^2^, respectively, a four order of magnitudes area change) considerably affects the gate current (associated to the amount of injected ionic charge into the organic polymers) and switching speeds (related to both the capacitance of the system and concentration and mobility of ionic charge carriers within the film).^[^ ^54^, ^55^, ^56^, ^57^, ^58^ ^]^ The difference in the relative improvement of switching performance between PEDOT:PSS‐based devices and the devices based on semiconducting polymers suggests that the lateral movement of ions inside the channel material, which is expected to be more prominent in PEDOT:PSS, plays and important role in the OECT switching behavior.^[^ ^57^ ^]^ In summary, these results clearly show that femtosecond laser patterning is a viable approach to isolate the conducting polymer channel from the rest of the polymer film, leading to a decrease in leakage current and faster switching speeds.

A significant advantage of this patterning method is the flexibility of changing the size and shape of the patterned and outlined areas for prototyping purposes. While the starting width and lengths of the drain and source electrodes are established during the initial step of gold contacts deposition (100 µm channel width, 10, 5, and 2.5 µm channel lengths), the selective removal of the insulating layer allows to tune the effective width of the electrodes connected to the active polymer. To demonstrate this, we fabricated an OECT with a channel width of 10 µm (channel length of 2.5 µm, Figure S8a, Supporting Information). Pushing the limits and using a single laser line to pattern the insulating layer, we show that it is possible to fabricate OECTs with an opening in the insulating layer of roughly 2 µm and a channel width of 5 µm (Figure S8b, Supporting Information).

### 2.2 Complementary Inverters

**Figure 3:**
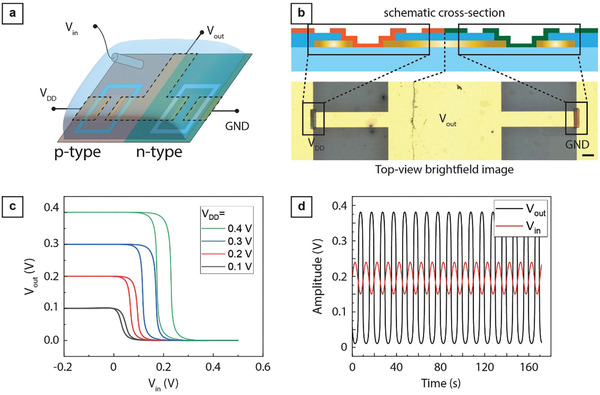
A femtosecond laser‐written logic gate‐inverter based on a p‐OECT and an n‐OECT. a) Schematic overview of the complementary inverter setup consisting of one p‐type and one n‐type OECT using Ag/AgCl pellet electrode as the input electrode and 0.1 M NaCl(aq) as the electrolyte. b) Schematic cross‐section (top) and brightfield image (bottom) of the inverter. c) Voltage transfer characteristics of the OECT‐based inverter at different supply voltages V_DD_. Scale bar, 100 µm. d) Oscillating voltage input and output of the inverter demonstrating amplification of the input signal at V_DD_ = 0.4 V.

Patterning polymers with p‐ and n‐type mode of transport allows to fabricate complementary logic circuits. In particular, OECT‐based inverters have been used to amplify low signals with limited power availability.^[^ ^15^ ^]^ As a proof of concept, we used ultrafast direct laser writing for the cleanroom‐free micropatterning of the p‐type PIBET‐AO polymer and the n‐type p(N‐T):PS10 polymer blend to obtain complementary inverter circuits (**Figure** **3**a,b). We used an Ag/AgCl pellet to control the input voltage (V_IN_), and a sodium chloride solution (0.1 M NaCl_(aq)_) as the electrolyte. Depending on the input voltage, either the PIBET‐AO or p(N‐T):PS10 OECT is in a conducting (ON) stage, resulting in an output voltage (V_OUT_) equal to the supply voltage (V_DD_) or the ground, respectively.

To spin‐coat PIBET‐AO on one half of the electrode sample and p(N‐T):PS10 on the other half, we used a tape masking approach and outlined the OECTs as previously described (see “Inverter Fabrication” in the Experimental Section; Figure S9, Supporting Information for details). Specifically, we used Kapton tape for masking since the tape is chemically stable and we could not observe glue residues after tape release (data not shown). We manufactured functional inverters in which a low voltage input signal (V_in_) is converted into a high voltage output signal (V_out_), and a high voltage input signal is converted into a low voltage output signal, resulting in a typical voltage transfer inverter characteristic curve (Figure 3c).

The inverters were further used as a push‐pull voltage amplifier. Using a sinus wave input voltage with an amplitude of 90 mV and a DC offset of 155 mV, we obtained an oscillating sinus wave output voltage with an amplitude of 400 mV (Figure 3d). To obtain a well‐balanced and optimized OECT‐based inverter, the conductivity of the individual channels could be further optimized by varying the thickness of the conjugated polymer layers.^[^ ^15^ ^]^ An inverter gate can be used as an amplification stage by selecting an operating point in the transition region between the ON and OFF state of the device. In fact, the gain defined as dV_out_/dV_in_ reaches its maximum value in the transition region and approaches 0 when the output is either “logical 0” or “logical 1”. Since the n‐type and p‐type transistors have threshold voltages that are opposite in sign, decreasing their absolute value would shrink the transition region and increase the gain. Moreover, matching the transconductance and the threshold voltages of the transistors would ensure that the inverter stage has the same transfer function for both ON‐to‐OFF and OFF‐to‐ON transitions, providing a constant gain at any operating point.

### 2.3 Enzymatic Biosensor for Glucose Monitoring

**Figure 4:**
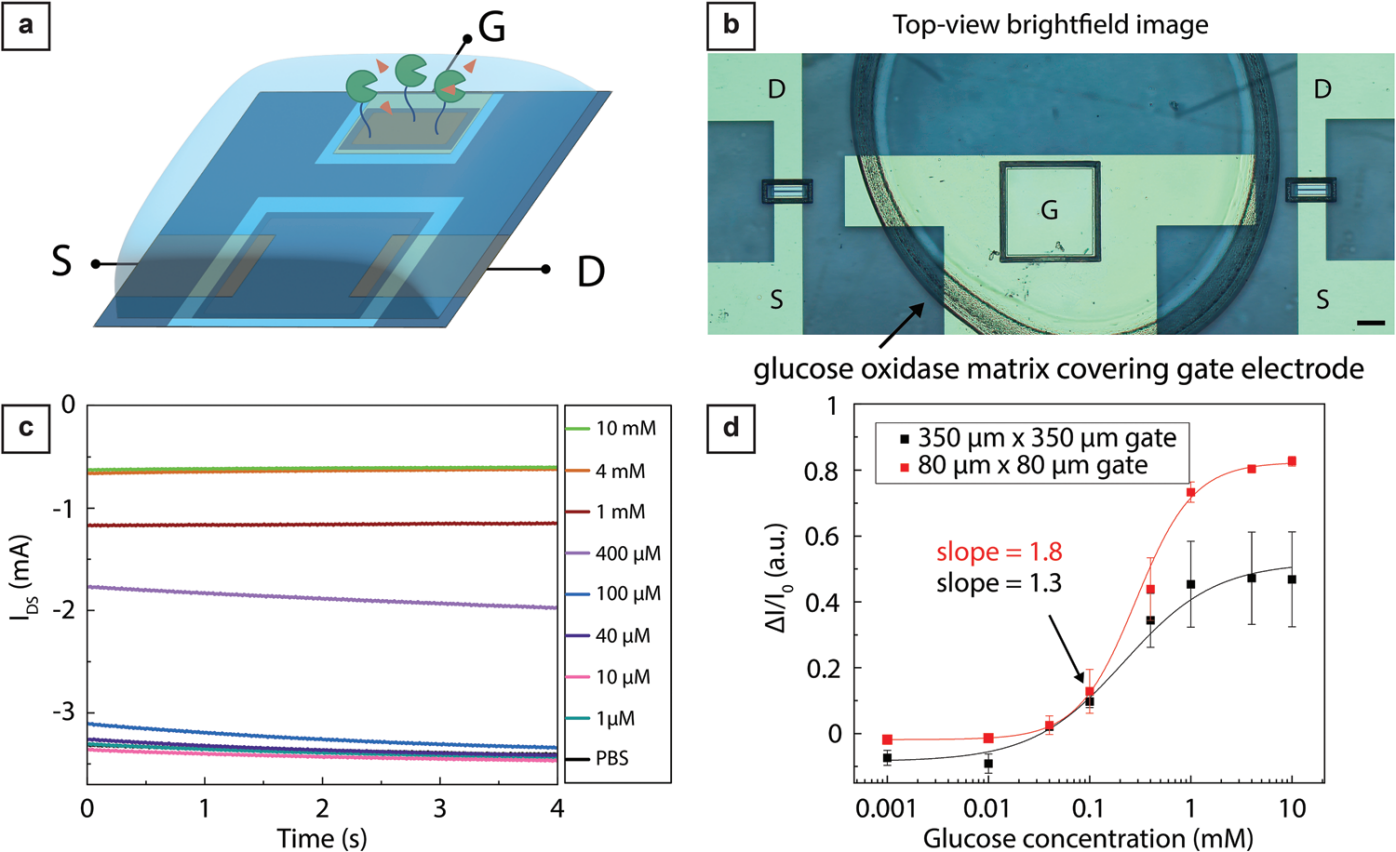
OECT‐based glucose sensing demonstrating how sensitivity depends on in‐plane gate size. a) Schematic illustration of the enzymatic OECT‐based glucose sensor using an in‐plane gate functionalized with glucose oxidase and 10 mM PBS buffer as electrolyte. b) Example (brightfield image) of the glucose sensor with glucose oxidase matrix on top of the gate electrode. In this image, the outlined area of PEDOT:PSS connected to the gate electrode was 350 µm × 350 µm. Scale, 100 µm. c) Example of amperometric measurements for different glucose concentrations in 10 mM PBS at V_G_ = −0.6 V and V_SD_ = −0.4 V using a PEDOT:PSS OECT with a channel area of 10 × 100 µm L x W and a gate electrode area of 80 µm × 80 µm. d) Glucose calibration curve with a sigmoidal logistic fit. The current measurement was averaged over two seconds for each concentration. Error bars show the standard error for three devices.

OECTs have shown great promise as biosensing devices due to their operation under physiological conditions, high signal amplification, and sensitivity.^[^ ^4^, ^55^, ^56^ ^]^ To demonstrate the versatility of our approach, we developed in‐plane enzyme‐based glucose sensors. In OECT‐based enzymatic biosensors, an enzyme is used as the biorecognition element, which is typically immobilized at the gate electrode. For the fabrication of this device type, we used ultrafast laser writing to micropattern both the channel (10 × 100 µm, L × W) and the in‐plane gate electrode (**Figure** **4**a,b; Videos S7 and S8, Supporting Information).

After patterning the insulating layer, we performed one spin‐coating step to deposit PEDOT:PSS at the channel and gate. We then outlined the channel and gate areas to isolate them from each other and separate them from the remaining portions of the film that do not participate in the device function. We further electrodeposited platinum nanoparticles on the gate electrode to enhance its electrocatalytic activity toward H_2_O_2_ oxidation (see experimental section for details).^[^ ^4^ ^]^ As a biorecognition element, we used the enzyme glucose oxidase, which generates H_2_O_2_ as a sub‐product of glucose oxidation. H_2_O_2_ oxidation changes the effective potential of the gate electrode, causing a large change in the source‐drain current in the OECT channel, thanks to the amplification effect of the OECT.^[^ ^60^, ^62^ ^]^ Here, glucose oxidase was immobilized on the gate electrode by embedding in glutaraldehyde‐crosslinked bovine serum albumin (BSA).^[^ ^4^ ^]^

We took advantage of the flexible design enabled by laser micropatterning to assess the impact of two different gate electrode areas, 350 µm × 350 µm and 80 µm × 80 µm, on biosensor characteristics.^[^ ^63^ ^]^ The performance and sensitivity of the OECT‐based biosensor were assessed by monitoring the relative changes in channel current for increasing glucose concentrations from 0.01 up to 10 mM (Figure 4c). We operated the device using 10 mM phosphate buffered saline (PBS) as an electrolyte and gate voltage of V_G_ = −0.6 V. After a short current spike, the drain‐source current quickly stabilized during device operation enabling us to limit the sampling period to 8 s. Normalized drain current response of the device shows that both sensors have a dynamic range within 100 µM–1 mM (Figure 4d; see Experimental Section and Figure S10, Supporting Information for details). This range is relevant for measuring glucose in human sweat, which ranges from 0.06 to 0.2 mM.^[^ ^64^ ^]^

The data also show that the smaller gate (i.e., larger channel/gate ratio) results in increased sensitivity with a slope value of 1.8 as opposed to a value of 1.3 for the large gate electrode (i.e., smaller channel/gate ratio) at 1 µM. These results align well with previously published data showing that larger channel/gate ratios result in increased device sensitivity to analytes interacting with the gate electrode.^[^ ^60^, ^63^, ^65^ ^]^ In the Faradaic regime, the effective potential that modulates the drain current is the sum of Faradaic and non‐Faradaic contributions, which are respectively dependent and independent of the concentration of the analyte. Since the non‐Faradaic contribution is inversely proportional to the channel/gate ratio, maximizing said ratio minimizes the non‐Faradaic contribution, making the concentration‐dependent term the dominating factor in the effective potential modulating the drain current, thus increasing the device sensitivity. The detection range is instead independent of the channel/gate ratio since both the background noise and the sensitivity are proportional to the channel/gate ratio.^[^ ^63^ ^]^ In contrast with previous methods for biosensor fabrication,^[^ ^63^ ^]^ ultrafast laser patterning would enable to easily modify the biosensor geometry further to match the desired application, such as the incorporation within integrated microfluidics sensing platforms.

## 3 Conclusions

In this work, we demonstrated that ultrafast femtosecond direct laser writing can be used to micropattern polymeric materials without needing a cleanroom environment, simplifying the fabrication of OECTs. We showed that, in contrast to previous methods using laser ablation for device fabrication,^[^ ^37^ ^]^ our approach, using focused ultrashort laser pulses, enables patterning with high resolution (down to 2 µm) without any observable damage of the surrounding polymer or layers underneath. We applied this method to pattern parylene C and polyimide, two insulating polymers commonly used as a passivation layer in OECT fabrication; demonstrating that the method is not limited by the type of polymer used for electrode passivation.

Moreover, we found that the process enables selective outlining of the OMIEC layer without damaging the insulating polymer layer underneath. Such selectivity is provided by carefully tuning the pulse energy of the laser, which, for the OMIEC layer, should be set 50% to 75% below the ablation threshold of the insulating polymer. We applied the method to outline three different OMIEC materials: a commercial conducting polymer (PEDOT:PSS), a p‐type organic semiconductor (PIBET‐AO), and an n‐type organic semiconducting blend (p(N‐T):PS10). Steady–state OECT device characteristics show that OECT maximum current and transconductance are preserved after the outlining step, confirming that using femtosecond pulsed laser writing to outline the OMIEC materials does not hamper the mixed ionic/electronic conductivity of the film. Gate current measurements from transfer characteristics and transient characteristics (i.e., ON/OFF switching times) show that outlining leads to OECTs with smaller leakage currents and faster switching times, particularly for the conducting PEDOT:PSS film, thereby verifying that the channel is indeed isolated from the rest of the polymer film.

Overall, OECT measurements show that the method can be used to outline different types of OMIEC materials independently on their chemistry or the solvent used for casting (water for PEDOT:PSS and chloroform:dichlorobenzene 10:1 for the organic semiconductors).

We further applied femtosecond pulsed laser writing to fabricate OECTs for two applications: OECT‐based logic inverters and OECT‐based enzymatic biosensors. Each device type possesses unique constraints in terms of design, namely the need for tightly spaced OECTs based on two different materials for inverters and the need for an in‐plane gate in biosensors. Data on device operation show that our approach is highly versatile and can be applied to the microfabrication of different device architectures, independently of the design constraints.

Our work shows a path for cleanroom‐free fabrication of OECTs. This method allows single‐digit micrometer resolution in polymer patterning and can be easily automated using machine vision. We expect that the advancements of cleanroom‐free methods for metal electrode deposition, such as through the use of stencil masks and conventional sputter coating tools,^[^ ^67^ ^]^ will, together with the method presented here, enable complete cleanroom‐free fabrication of OECTs and an even faster route for the prototyping and scaling of microscale devices for bioelectronics.

## 4 Experimental Section

### Materials and Reagents

Chloroform (product number 366 927), 1,2‐Dichlorobenzene (product number 240 664), Ethylene glycol (product number 102 466), 4‐Bromoanisole (product number B56501), Triton X‐100 (product number T8787), Poly(vinyl alcohol) (M_W_ 31–50 k product number 363 073), Sodium Chloride (product number S5886), Glucose Oxidase (product number G7141), D‐(+)‐Glucose (product number G8270), Glutaraldehyde solution (product number 340 855), 3‐(Trimethoxysilyl)propyl methacrylate (A174, product number 440 159), Chloroplatinic acid hexahydrate (H_2_PtCl_2_•6H_2_0, product number 206 083), Sulfuric acid (H_2_SO_4_), Bovine Serum Albumin (product number A3059), 4‐dodecylbenzenesulfonic acid (DBSA, product number 44 198)) and polystyrene (10 kDa, product number 81 406) were purchased from Merck. GOPS (product number A18431) and PBS (product number 18912‐014) were purchased from Thermo Fisher Scientific. PEDOT:PSS was purchased by Merck (product number 655 201) or by Heareaus (product name Clevios PH 1000). Polyimide precursor solution (PI2545) was purchased from HD MicroSystems. p(N‐T) and PIBET‐AO were synthesized as previously described.^[^ ^13^, ^14^ ^]^ Blends of p(N‐T) and polystyrene 10 KDa (PS10) were prepared by mixing stock solutions of p(N‐T) (15 g L^−1^) and PS10 (50 g L^−1^) to reach a final concentration of 10 g L^−1^ for p(N‐T) and 4.8 g L^−1^ for PS10.

### Gold Electrode Fabrication and Insulating Layer Deposition

Borosilicate glass wafers were coated with standard lift‐off photoresist and exposed using Heidelberg Instruments GmbH MLA150. After development, an electron beam evaporator Provac PAK 600 Coating System was used to deposit 10 nm of chromium as adhesion layer and 100 nm of gold to create the contacts. After deposition, propylene glycol monomethyl ether acetate (PGMEA, purchased as RER 60, Fujifilm, US) was used to remove any trace of photoresist, followed by deionized water rinse and nitrogen drying.

It was used alternatively parylene or polyimide as insulation layer. For Parylene C, the samples were immersed in 5% v/v A174‐silane, 1:1 mixture of isopropyl alcohol:deionized water for 15 min and dried with an air gun before the Parylene C deposition. A PDS 2010 Labcoater 2 (SCS, UK) was used to deposit a parylene layer between 0.5 and 2 µm. Alternatively, a 1.5 µm polyimide insulating layer was deposited by spin‐coating 200 µl of a commercial solution polyamic acid in an N‐methyl‐2‐pyrrolidone (PI2545, HD MicroSystems) at 5000 rmp for 60 s and then baking at 250 °C for 30 min for conversion to polyimide. In the presented data, polyimide was used as the insulating layer for PIBET‐AO and p(N‐T) OECTs and parylene C for the remaining devices.

### OECT Manufacturing by Direct Femtosecond Laser Writing

A commercial laser lithography system (Photonics Professional GT2, Nanoscribe, Germany) with femtosecond laser radiation (780 nm wavelength, 80–120 fs pulse duration, 80 MHz repetition rate) and a 20x air immersion objective (Plan‐Apochromat 20x/0.8 440640–9903, Zeiss, Germany) was used to pattern the insulating layer (composed of parylene or polyimide). Arrays of lines with a distance of 250 nm were used to open up a window in the insulating layer with an area of 120 µm × 30 µm for the OECT channel above the source‐drain contacts. The laser settings were set to a speed of 1 mm s^−1^ and a pulse energy of 600 pJ. To remove the residue of the insulating polymer after laser patterning, the samples were air plasma treated (FEMTO, Diener electronics) at 70 W power and 0.2 mbar pressure for 2 × 4 min to remove the remaining debris from the laser writing.

OECTs were then fabricated by spin‐coating the different conjugated polymer solutions on top of the samples with patterned insulating layer: 1) PEDOT:PSS with 5% (v/v) ethylene glycol, 0.25% (v/v) triton x‐100 and 0.25% (v/v) GOPS, 2) PIBET‐AO (10 mg mL^−1^) in 10:1 chloroform:1,2‐Dichlorobenzene with 5% (v/v) 4‐Bromoanisole, and 3) p(N‐T) (10 mg mL^−1^) blended with 10 k polystyrene (1:6 ratio of monomer Mw) in 10:1 chloroform:1,2‐Dichlorobenzene with 5% (v/v) 4‐Bromoanisole. PEDOT:PSS, PIBET‐AO, and p(N‐T):PS10 were respectively spin‐coated at 4000, 2500, and 1000 rpm for 60 s to decrease gold/polymer contact resistance and improve both charge transport from bulk gold to interface and charge injection from gold into conjugated polymers.^[^ ^68^ ^]^ PEDOT:PSS OECTs were kept in the oven at 130 °C for 30 min, and PIBET‐AO and p(N‐T):PS10 for 10 min.

Laser exposure was again used to scribe the conjugated polymer to outline and isolate the channel area of the OECT from the remaining polymer film. A 15‐µm‐wide rectangle consisting of 20 lines spaced by 750 nm was drawn around the channel area with a pulse energy between 75 and 300 pJ and a speed between 25 and 150 mm s^−1^.

Since the insulating polymers are more chemically and thermally stable than most conjugated polymers, regeneration of the substrate can be achieved by mild dry or wet etching of the conjugated polymer (for example, using oxygen plasma or basic piranha solution made of H_2_O:H_2_O_2_:NH_3_ 5:1:1), enabling the reuse of the patterned electrodes.

### Inverter Fabrication

The approach described in fabricating OECTs was adapted to obtain inverters. Specifically, to achieve selective deposition of the n‐type p(N‐T):PS10 and p‐type PIBET‐AO polymers, a tape masking approach with an additional sacrificial poly(vinyl alcohol) (PVA) layer was used (Figure S9, Supporting Information). First, Kapton tape was used to mask one half of the inverter, and a 5% solution of PVA in water was spin‐coated at 2000 rpm for 60 s and left to dry at room temperature for 10 min. When the PVA layer was dry, the Kapton tape was removed, and the first conjugated polymer solution was spin‐coated and baked, as described above. The sacrificial PVA layer was dissolved in water under shaking for 5 min, removing the conjugated polymer on top of the PVA layer and obtaining an evenly spin‐coated conjugated polymer film on one‐half of the electrode sample. The procedure with Kapton tape and spin‐coated PVA was repeated on the other half of the sample using the second conjugated polymer solution. Combining Kapton tape masking and the sacrificial PVA layer allows to generate an evenly spin‐coated conjugated polymer film. Kapton tape has a thickness of 30 µm, leading to uneven deposition at the tape edge. In contrast, the thinner PVA layer (≈1 µm) enables even deposition without the insurgence of edge effects.

### Glucose Sensor Fabrication

The enzymatic biosensor device was fabricated using the protocol described for OECTs with the following modifications. A different PEDOT:PSS formulation with increased stability was used: PEDOT:PSS with 5% (v/v) ethylene glycol, 0.25% (v/v) DBSA, and 1% (v/v) GOPS.^[^ ^69^ ^]^ To fabricate an in‐plane gate electrode, a 350 µm × 350 µm or 80 µm × 80 µm area of the insulating layer was removed by laser scribing to expose an additional gold area. After spin‐coating and baking the conducting polymer, the gate electrode was outlined by a 15 µm wide square, with the same parameters used to outline the channel area. As previously reported, the gate electrode was then functionalized with Pt nanoparticles in aqueous 5 mM H_2_PtCl_6,_ 50 mM H_2_SO_4_ through electrochemical deposition using the gate as working electrode.^[^ ^4^ ^]^ Using a potentiostat (Autolab PGSTAT204) a potential of 0.7 V was applied for 10 s, followed by −0.2 V for 15 s.

To immobilize the enzyme, a solution of 1.5% w/v BSA, 1.5% w/v glucose oxidase, and 1% w/v glutaraldehyde in water was freshly mixed, and a droplet of 100 nL was deposited on top of the gate electrode.^[^ ^66^ ^]^ Once the droplet had dried out and the BSA matrix was cross‐linked, the sample was rinsed with water before being used for the sensing measurements.

### Electrical Measurements of Gate and Drain‐Source Currents and Switching Performance of the Devices

A Keithley 4200A‐SCS parameter analyzer (Tektronix, USA) equipped with two source measurement units (SMU) and one pulse measurement unit (PMU) was used for characterizing OECTs, inverters, and glucose sensors.

OECTs and inverter measurements were performed using 0.1 M NaCl_(aq)_ solution as the electrolyte and a silver/silver‐chloride (Ag/AgCl) pellet as the gate electrode. V_G_‐I_DS_ transfer characteristics were measured using the two SMUs, and the ON/OFF device switching was measured with the PMU. ON and OFF switching times were calculated by the time it took to reach 90% of the maximum current and 10% of the maximum drain current, respectively. For the inverter setup, the Ag/AgCl pellet was used to apply V_in_, and an additional Keithley 2410 was connected to supply the V_DD_ potential.

The glucose sensing measurements were performed using 10 mM PBS as an electrolyte solution, and the fabricated in‐plane gate. A glucose stock solution of 100 mM in PBS was prepared and serially diluted in 10 mM PBS to obtain the different concentrations. For the current measurements, a volume of 100 µL glucose solution was added on the device covering the gate and the channel electrodes, and a gate voltage of V_G_ = −0.6 V was applied for eight seconds with V_D_ = −0.4 V. The response for each concentration was determined by averaging the drain current value of the last two seconds. After one measurement, the solution was removed, and a new higher concentration was added on the same device. The glucose calibration curve was calculated by normalizing the drain current using the equation:

(1) $$\frac{\Delta I}{I_{0}}=\frac{I_{c}-I_{0}}{I_{0}}$$

where *I_c_* is the drain current at the specific glucose concentration and *I*_0_ is the drain current in 10 mM PBS. The dose‐response curve was then fitted to a logistic sigmoidal function using Origin:

(2) $$f\left(x\right)=\frac{A_{1}-A_{2}}{1+{\left(\frac{x}{x_{0}}\right)}^{P}}+A_{2}$$

### High Speed Imaging of the Electrochromic Effect during OECT Switching

Video recordings of the ON/OFF switching of PEDOT:PSS‐based and PIBET‐AO‐based OECTs were performed using a Phantom Miro M120 high‐speed camera (Visio Research/ AMETEK, USA) at a frame rate of 10 k Hz or 13 kHz, with an upright Zeiss microscope and a 40x objective. For the video analysis, Fiji was used to select a region of interest and plot the mean gray value over time.

### Statistical Analysis

Origin Pro was used to calculate p‐values using two‐sample t‐tests.

## Conflict of Interest

The authors declare no conflict of interest.

## Supporting information

Supporting Information

Supplemental Video 1

Supplemental Video 2

Supplemental Video 3

Supplemental Video 4

Supplemental Video 5

Supplemental Video 6

Supplemental Video 7

Supplemental Video 8

## Data Availability

The data that support the findings of this study are available from the corresponding author upon reasonable request.
