# Supplementary material for: Cleanroom‐Free Direct Laser Micropatterning of Polymers for Organic Electrochemical Transistors in Logic Circuits and Glucose Biosensors
Source: Adv Sci (Weinh). 2024 Jan 15;11(27):2307042. doi: 10.1002/advs.202307042 (PMC11251563; doi:10.1002/advs.202307042)
Supplement: Supplementary file 1 — Supporting Information [file ADVS-11-2307042-s009.pdf]

ADVANCED SCIENCE
Open Access

Supporting Information

for *Adv. Sci.*, DOI 10.1002/advs.202307042

Cleanroom-Free Direct Laser Micropatterning of Polymers for Organic Electrochemical Transistors in Logic Circuits and Glucose Biosensors

Alessandro Enrico, Sebastian Buchmann, Fabio De Ferrari, Yunfan Lin, Yazhou Wang, Wan Yue, Gustaf Mårtensson, Göran Stemme, Mahiar Max Hamedi, Frank Niklaus, Anna Herland* and Erica Zeglio**

Supporting Information

Cleanroom-free direct laser micropatterning of polymers for organic electrochemical transistors in logic circuits and glucose biosensors

Alessandro Enrico[1,2,§], Sebastian Buchmann[3,4,§], Fabio De Ferrari[1], Yunfan Lin[3], Yazhou Wang[5], Wan Yue[6], Gustaf Mårtensson[3,7], Göran Stemme[1], Mahiar Max Hamedi[8], Frank Niklaus[1,], Anna Herland[3,5,*], and Erica Zeglio[4,9,*]*

1: A. Enrico, F. De Ferrari, G. Stemme, F. Niklaus
Department of Micro and Nanosystems, KTH Royal Institute of Technology, Malvinas väg 10, 100 44 Stockholm, Sweden.
*E-mail: frank@kth.se
2: A. Enrico
Synthetic Physiology lab, Department of Civil Engineering and Architecture, University of Pavia, Via Ferrata 3, 27100, Pavia, Italy.
3: S. Buchmann, Y. Lin, G. Mårtensson, A. Herland
Division of Nanobiotechnology, KTH Royal Institute of Technology, Tomtebodavägen 23a 171 65 Solna, Sweden.
4: S. Buchmann, A. Herland, E. Zeglio
AIMES – Center for the Advancement of Integrated Medical and Engineering Sciences, Department of Neuroscience, Karolinska Institute, 17177 Stockholm, Sweden.
*E-mail: anna.herland@ki.se
5: Y. Wang
Guangzhou Key Laboratory of Flexible Electronic Materials and Wearable Devices, School of Materials Science and Engineering, Sun Yat-sen University, Guangzhou 510275, People's Republic of China.
6: W. Yue
Key Laboratory for Polymeric Composite and Functional Materials of Ministry of Education, School of Materials Science and Engineering, Sun Yat-sen University, Guangzhou 510275, People's Republic of China.
7: G. Mårtensson
Mycronic AB, Täby, Sweden.

8: M. M. Hamedi
Department of Fibre and Polymer Technology, School of Engineering Sciences in Chemistry, Biotechnology and Health, KTH Royal Institute of Technology, Teknikringen 56, 10044 Stockholm, Sweden.
9: E. Zeglio
Wallenberg Initiative Materials Science for Sustainability, Department of Materials and Environmental Chemistry, Stockholm University, 114 18 Stockholm, Sweden.
*E-mail: erica.zeglio@mmk.su.se

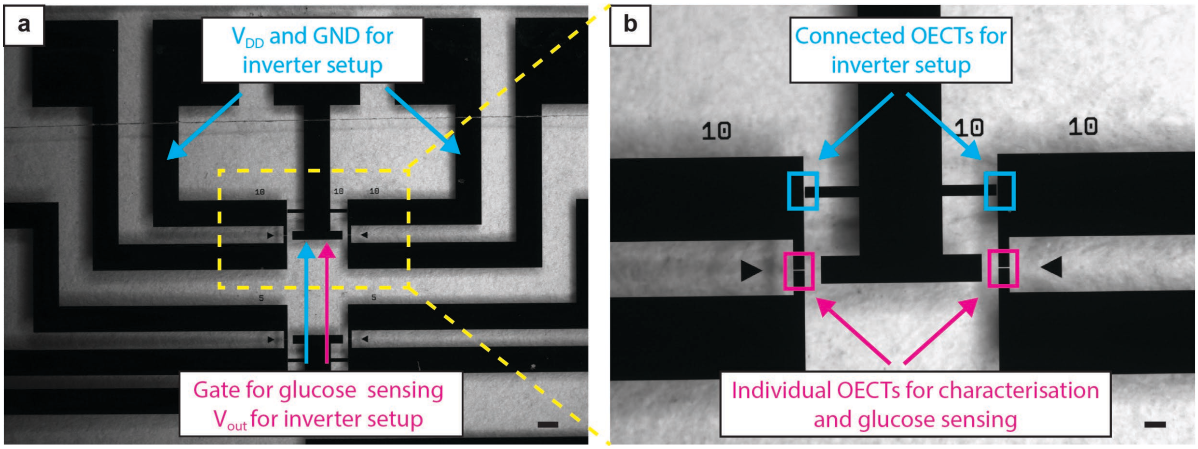

Figure S1 - Gold electrode design overview. a) Depending on the laser patterning, the electrode configuration can be used to build a biosensor or an inverter. b) Zoomed-in images highlight how the electrode layout can be used to fabricate OECTs (external or in-plane gates) or OECTs-based inverters. The electrode separations in both images are 10 µm. Scale bars, 500 µm in a) and 200 µm in b).

Video S1 - Removal of parylene C to expose the source and drain electrodes. Electrode separation 10 µm.

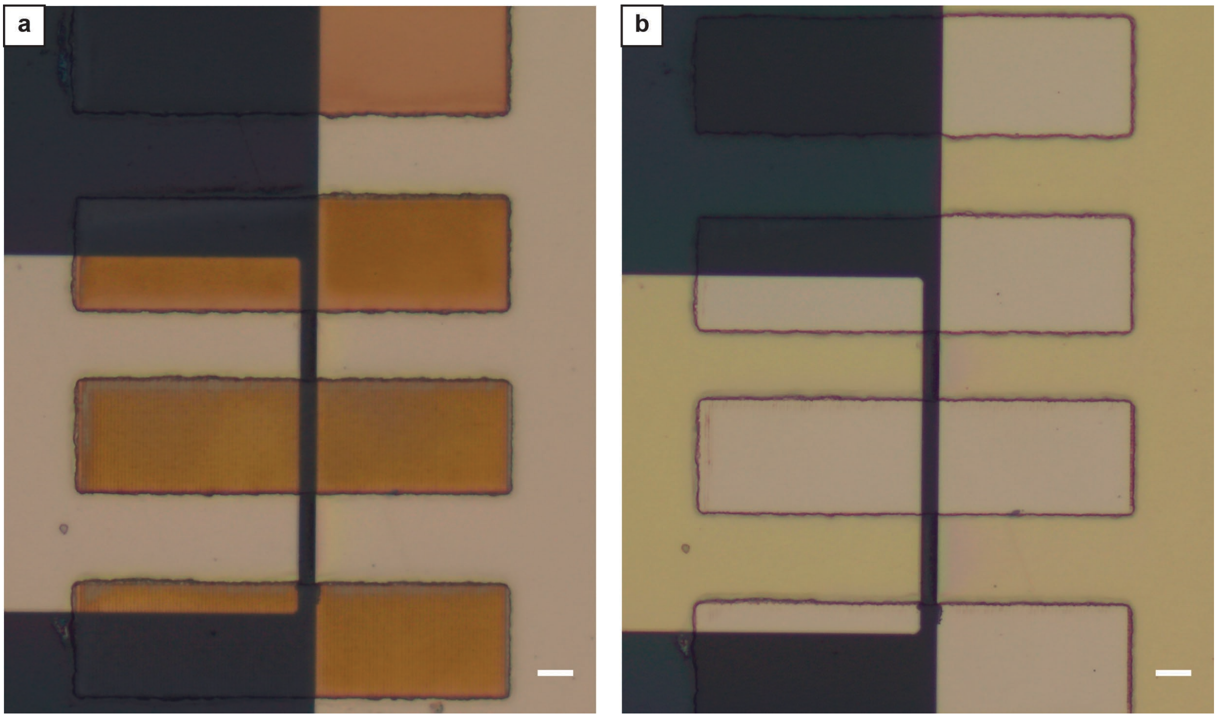

Figure S2 – Polymer residue removal with plasma cleaning. Brightfield (top-view) images of laser-patterned areas a) before and b) after 8 minutes of plasma cleaning. Scale bars, 10 µm.

Video S2 - Outlining of the PEDOT:PSS polymer area contributing to the OECT channel performance. Electrode separation 10 µm.

Table S1 - Summary of device parameters for PEDOT:PSS from transfer curves in Figure 2c and d, with V_{DS} = -0.6 V.

**Polymer**	**Channel length (µm)**	**Max. I_{DS} (mA)**	**Max. g_m (mS)**
PEDOT:PSS outlined	10	-5.6 ± 0.5	8.5 ± 0.8
	5	-9.4 ± 0.4	13.9 ± 0.6
	2.5	-12.1 ± 0.4	17.8 ± 0.5
PEDOT:PSS non-outlined	10	-6.8 ± 0.9	10.3 ± 1.3
	5	-8.2 ± 0.9	11.5 ± 1.3
	2.5	-13.5 ± 0.3	20.2 ± 0.5

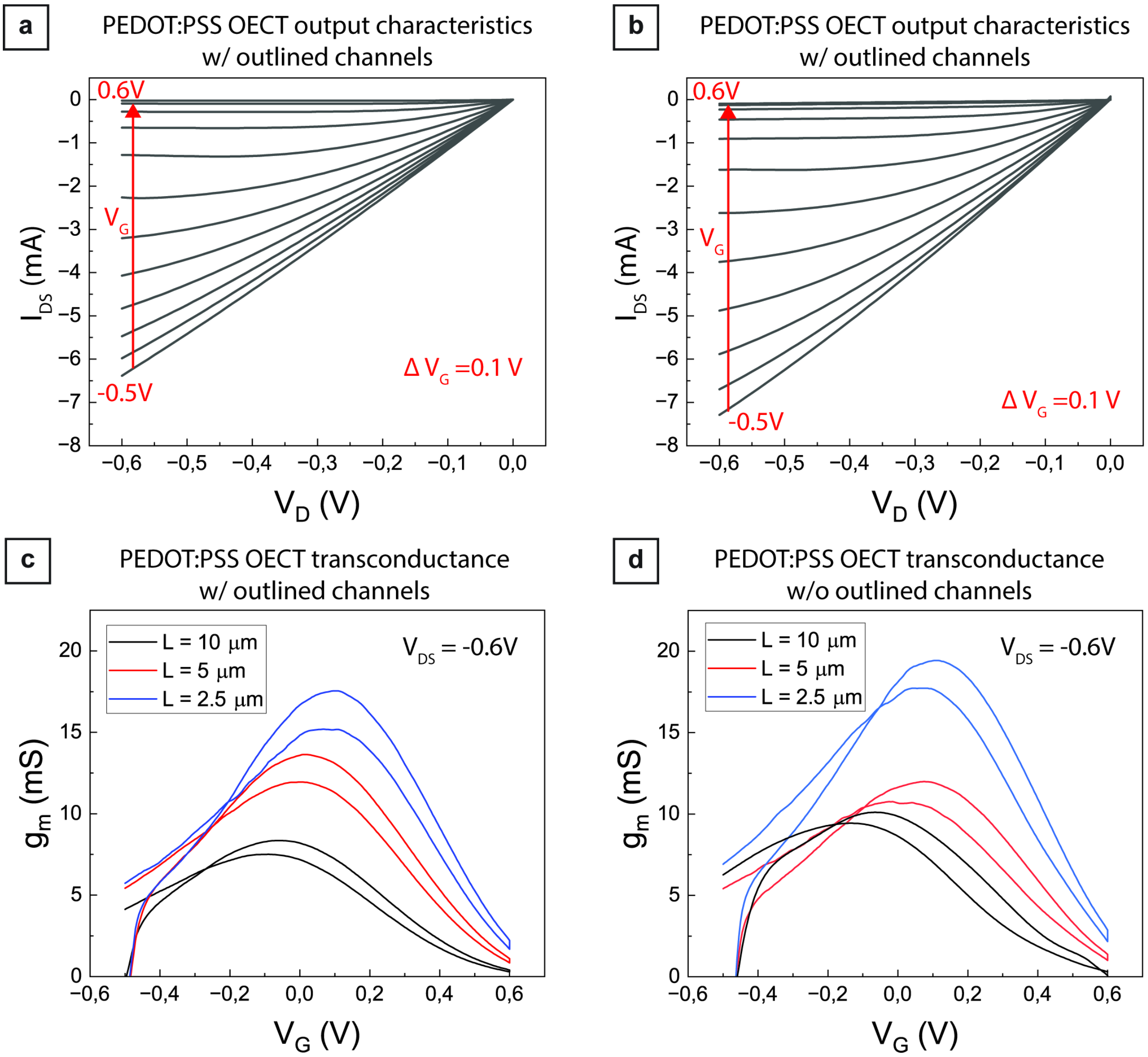

Figure S3 - PEDOT:PSS OECT performances. a) Representative output characteristics for outlined PEDOT:PSS OECTs with channel length 10 µm. b) Representative output characteristics for non-outlined PEDOT:PSS OECTs with channel length 10 µm. c) and d) show the average transconductance (g_m) of five outlined and non-outlined PEDOT:PSS OECTs with channel lengths of 10, 5, and 2.5 µm, respectively. The derivative was taken from the average transfer curves shown in Figures 2c-d to obtain the transconductance. Ten measurement points were averaged to smoothen the curve.

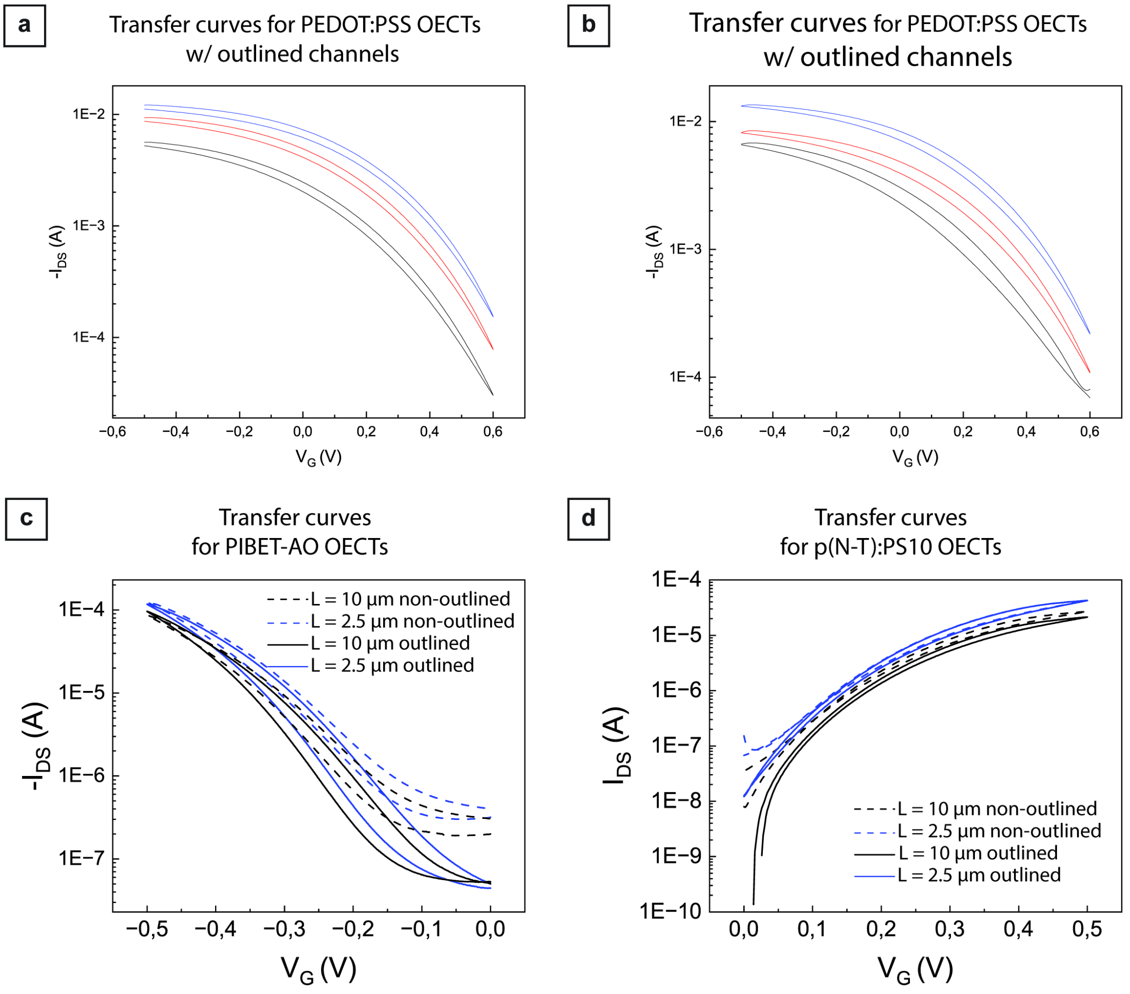

Figure S4 - Transfer functions of the fabricated OECT devices in log scale.

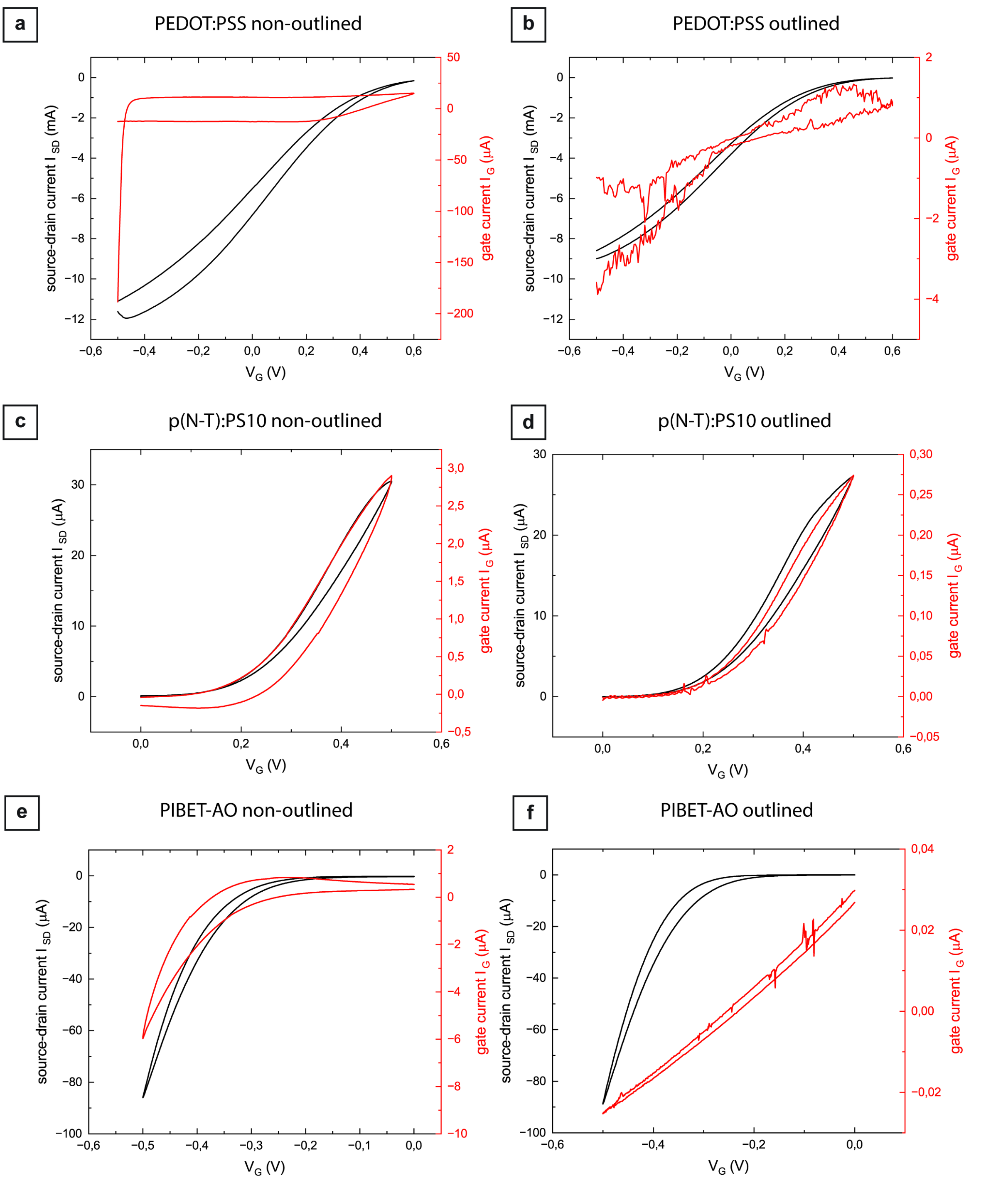

Figure S5. Transfer curve with related gate currents. a) Non-outlined and b) outlined PEDOT:PSS OECTs. Outlining the channel area decreases the maximum gate leakage current (from -150 µA range to -3µA using V_{DS} = -0.6V; see Table S2 for detailed values) of the resulting OECTs. c)-d) Transfer curve for outlined and non-outlined and p(N-T):PS10 OECTs with V_{DS} = 0.5V. Maximum gate current decreases from 3 µA to 0.3 µA. e)-f) Transfer curve for outlined and non-outlined and PIBET-AO OECTs with V_{DS} = 0.5V. Maximum gate current decreases from -5 µA to -0.02 µA.

Table S2 - Overview of the average maximum gate current for outlined and not outlined OECTs with a channel length of 10 µm from the transfer measurements shown in Figure 2.

Polymer	Outlined OECTs $I_{G,max}$ (µA)	Non-outlined OECTs $I_{G,max}$ (µA)
PEDOT:PSS	-2 ± 1	-151 ± 42
p(N-T):PS10	0.14 ± 0.06	2.6 ± 0.2
PIBET-AO	-0.40 ± 0.36	-6.2 ± 0.2

Table S3 - Summary of device parameters for PIBET-AO and p(N-T):PS10 from transfer curves in Figure 2h-i, with V_{DS} = -0.5 V and 0.5V, respectively.

**Polymer**	**Channel length (µm)**	**Max. I_{DS} (µA)**	**Max. *g* (µS)**
PIBET-AO outlined	10	-96 ± 5	1100 ± 60
	2.5	-118 ± 19	1230 ± 200
PIBET-AO non-outlined	10	-86 ± 6	856 ± 75
	2.5	-129 ± 5	1610 ± 170
p(N-T):PS10 outlined	10	21 ± 2	108 ± 8
	2.5	43 ± 9	246 ± 49
p(N-T):PS10 non-outlined	10	29 ± 1	147 ± 9
	2.5	43 ± 6	230 ± 31

Video S3 - Switching behavior of non-outlined PEDOT:PSS devices. Three switching events were recorded. The video speed has been slowed down 20 times.

Video S4 - Switching behavior of outlined PEDOT:PSS devices. Four switching events were recorded. The video speed has been slowed down 20 times.

Video S5 - Switching behavior of non-outlined PIBET-AO devices. Two switching events were recorded. The video speed has been slowed down 20 times.

Video S6 - Switching behavior of outlined PIBET-AO devices. One switching event was recorded. The video speed has been slowed down 20 times.

Video S7 - Removal of parylene C to expose the in-plane gate electrode. 65 µm edge.

Video S8 - Outlining of the PEDOT:PSS polymer area contributing to the active area of the in-plane gate electrode performance. 80 µm edge.

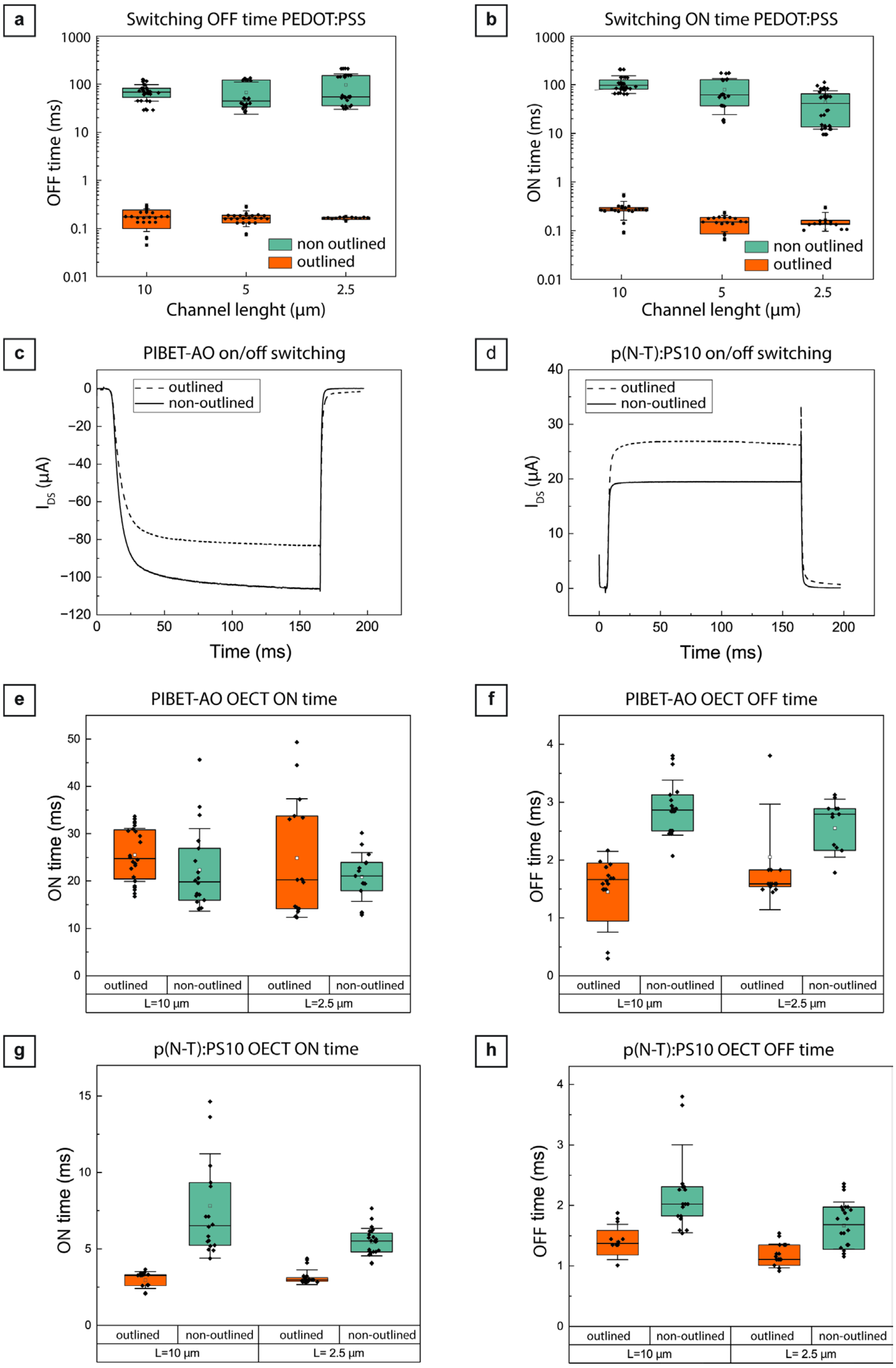

Figure S6 – Detailed OECT switching behaviors. a) Switching OFF and b) ON time constants of outlined and non-outlined PEDOT:PSS OECTs for different channel lengths. c) ON/OFF switching characteristics of an outlined and non-outlined PIBET-AO OECT with V_{DS} = -0.5 V

and V_G switching from 0 V to -0.5 V. d) ON/OFF switching characteristics of an outlined and non-outlined p(N-T):PS10 OECT with V_{DS} = 0.5 V and V_G switching from 0 V to 0.5 V. Switching time constants of e-f) PIBET-AO-based and g)-h) p(N-T):PS10-based OECTs. The switching time is defined as the interval necessary for the transistor voltage output to sweep from 10% to 90% of the full dynamics (ON to OFF state) of the voltage output. The displayed data in e)-h) using boxplot notation refer to OECTs with drain-source electrode width of 100 μm, two different electrode separations L (2.5 and 10 μm), channel thickness around 100 nm, and with or without restricting the portion of semiconductive polymer contributing to the conduction to the active channel area. Each dot in the box plot represents the duration of a single switching event. Error bars show the standard deviations, with the centerline being the median value, and box plots corresponding to 25 and 75 percentiles.

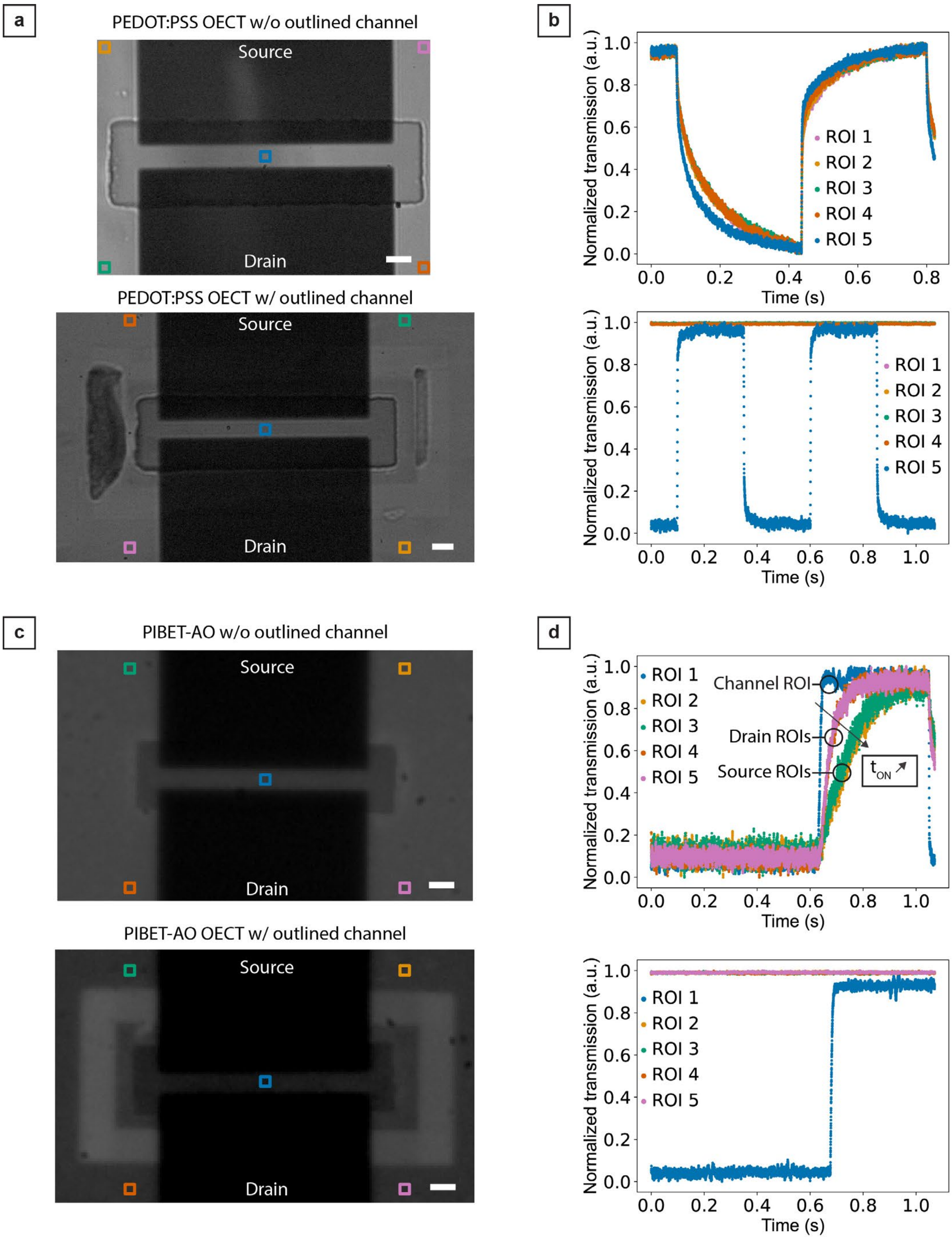

Figure S7 - High-speed of the polymer doping and de-doping in a PEDOT:PSS OECT (a and b) and a PIBET-AO OECT (c and d). We first collected the transmitted signal through the polymer in the regions of interest (ROIs) between the electrodes (in blue) and on the side of the OECTs (in red). To compare the signal change between different ROIs, the signal values of each ROI was plotted between "0" and "1". The "0" baseline is the low-transmission signal obtained by subtracting the minimum value from the signal values for each ROIs. This rescaled signal was then normalized with respect to the rescaled maximum signal value for each ROI to obtain the "1" high-transmission level. Doping and

dedoping occur in the entire film for unpatterned devices (a and c), and only in the outlined channels for patterned ones (b and d). Scale bars, 10 µm.

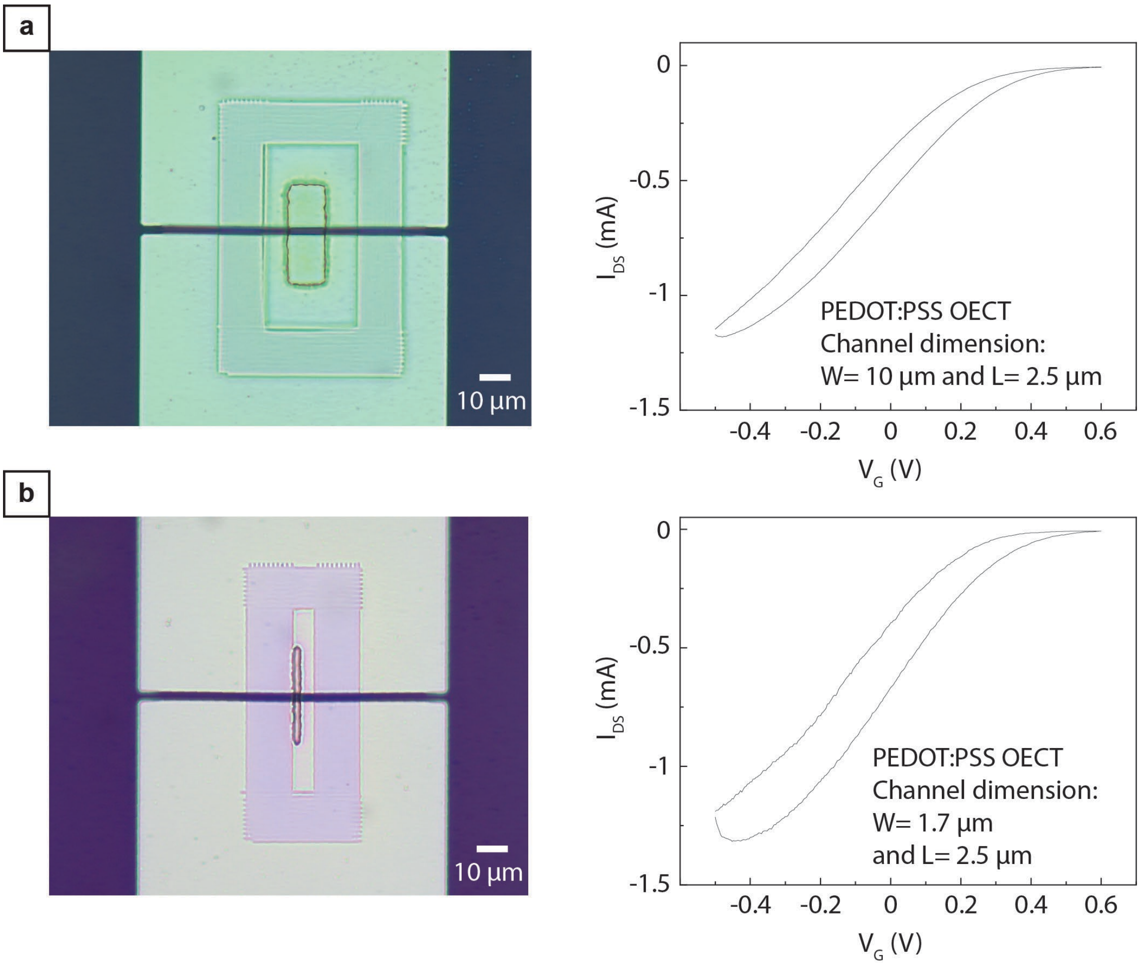

Figure S8 - Direct laser writing allows fast prototyping of different channel geometries with single-digit micrometer resolution. Brightfield images (on the left) and transfer curves (on the right) for PEDOT:PSS OECTs with different channel geometries. The openings in the insulating polymer are 30 µm x 10 µm for a), and 30 µm x 2 µm for b). These results illustrate how OECTs with varying channel widths can be fabricated by simply changing the size of the laser-patterned window in the insulating layer. b) By illuminating the insulating layer with a single-line design, we could fabricate OECTs with an inner channel width of 1.7 ± 0.1 µm, demonstrating the resolution capabilities of this method to microfabricate functional OECTs.

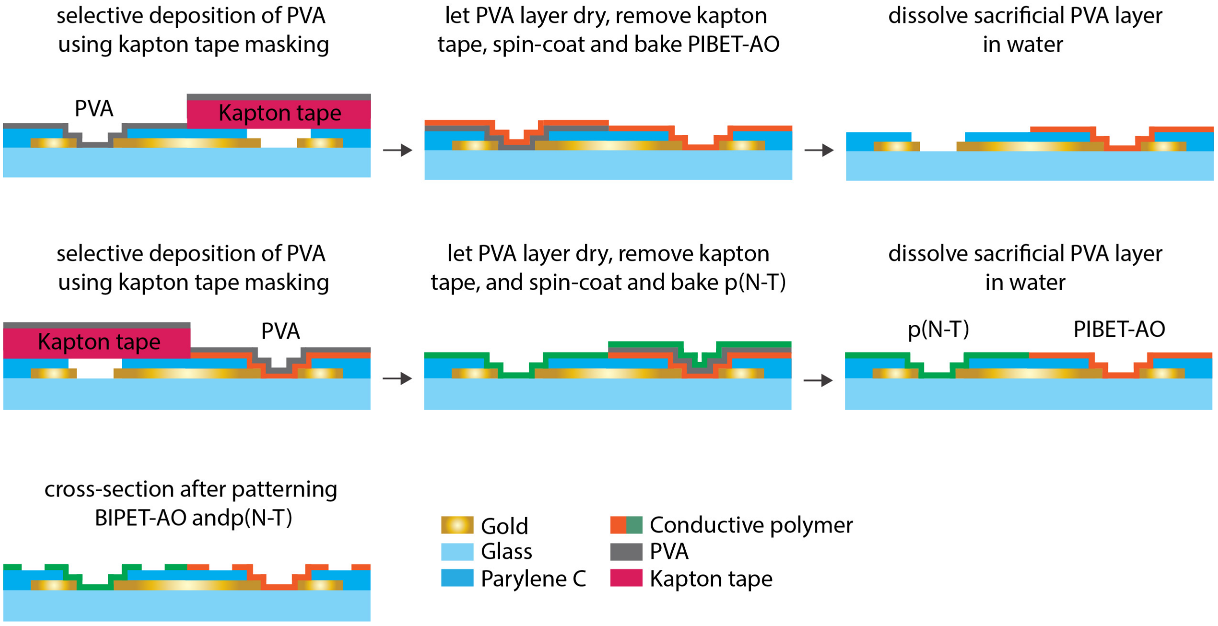

Figure S9 - Schematic illustration of the fabrication approach to obtain an inverter. The device consists of two transistors whose channel materials are PIBET-AO and p(N-T):PS10 as p-type and n-type polymers, respectively. The PVA layer is first spin-coated and let dry at room temperature and atmosphere for 10 minutes. Then, Kapton tape is removed, and the conjugated polymer of choice is spin-coated and subsequently annealed. The process is then repeated on the other half of the device with the complementary polymer.

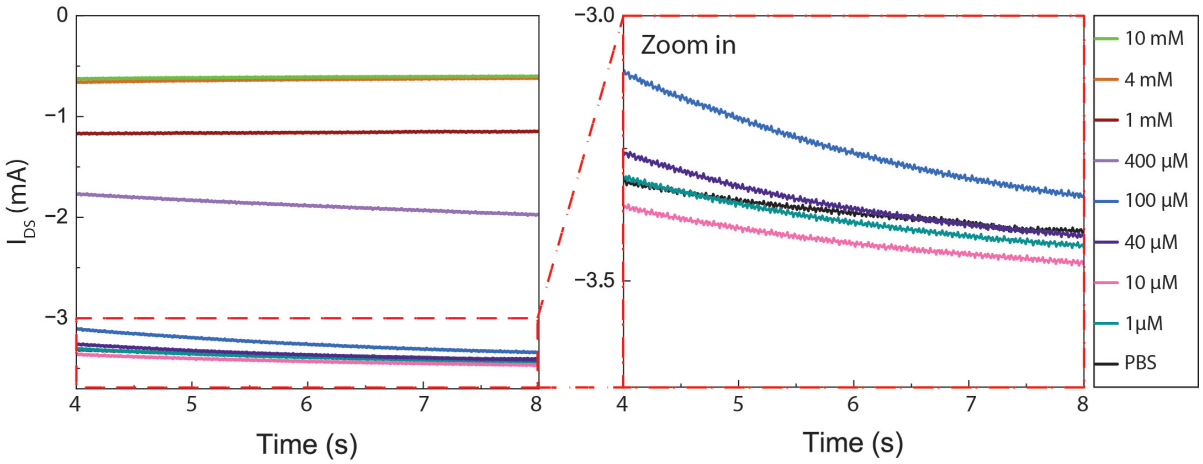

Figure S10 - OECT-based glucose sensing. Example of amperometric measurements with zoom-in window for low glucose concentrations in 10 mM PBS at V_G = -0.6 V and V_D = -0.4 V. The PEDOT:PSS OECT had a channel area of 10 x 100 µm L x W and a gate electrode area of 80 µm x 80 µm.
